# Automated Remodelling of Connectors in Fixed Partial Dentures

**DOI:** 10.3390/dj11110252

**Published:** 2023-10-27

**Authors:** Hassen Jemaa, Michael Eisenburger, Andreas Greuling

**Affiliations:** Department of Prosthetic Dentistry and Biomedical Materials Science, Hannover Medical School, Carl-Neuberg-Straße 1, 30625 Hannover, Germany; jemaa.hassen@mh-hannover.de (H.J.); eisenburger.michael@mh-hannover.de (M.E.)

**Keywords:** fixed partial denture, connector, finite element analysis, parametric modelling, dental implants

## Abstract

In this study, an approach for automated parametric remodelling of the connector cross-sectional area in a CAD model of a given fixed partial denture (FPD) geometry was developed and then applied to a 4-unit FPD. The remodelling algorithm was implemented using Rhinoceros and the Grasshopper plugin. The generated CAD models were used to perform a finite element analysis with Ansys to analyse the stress distribution in an implant-supported 4-unit FPD for different connector designs. The results showed that the type of connector adjustment matters and that the resulting stress can be significantly different even for connectors with the same cross-sectional area. For tensile stresses, a reduction in the connector cross-sectional area from the gingival side showed the highest influence on each connector type. It can be concluded that the developed algorithm is suitable for automatic connector detection and adjustment.

## 1. Introduction

In dentistry, a fixed partial denture (FPD) is often used to replace one or more missing teeth. Metal–ceramic FPDs have been used successfully for decades and have been reported with a 5-year survival of 94.4% in a systematic review by Sailer et al. [1]. Nowadays, various ceramic-based materials are used for FPDs, whereas da Silva et al. reported the main advantages and disadvantages of different ceramic systems and processing methods [2]. Among those alternatives, zirconia-based FPDs are promising, either with a zirconia-based framework and a veneering or in a monolithic form. For veneered zirconia-based 3-unit FPDs, Zarone et al. recently reported a 14-year survival of 91% for patients wearing an FPD [3]. According to a recent review by Laumbacher et al., veneering fractures are the predominant problem for tooth-supported as well as implant-supported crowns and multi-unit FPDs [4]. For monolithic zirconia-based FPDs, a recent review by Kim et al. found high 5-year cumulative survival rates of 99.60%, indicating an excellent 5-year performance [5]. Longer follow-up periods are currently missing.

In addition to the choice of material, the connector design also plays an important role in the fracture load, which has been shown in many in vitro studies for 3-unit and 4-unit FPDs [6,7,8,9,10,11]. Larsson et al. [7] for instance found that the fracture strength was higher for a large connector diameter. Oh et al. found that the radius of the connectors has an influence, and suggested that the radius at the gingival site plays a prominent role, while the curvature at the occlusal site can be as sharp as needed for the aesthetics [6].

Besides the in vitro studies mentioned above, finite element analysis (FEA) was used in the literature to investigate the effect of connector design on the stresses in FPDs, and many studies found the connector design to have a significant influence on the failure behaviour or on observed stresses [12,13,14,15]. Furthermore, Alberto et al. [12] found the radius of the interdental gingival shape to have a significant influence on the stresses in FPDs, whereas the smallest radius led to the highest tensile stresses. Almasi et al. performed an FEA on 3-unit FPDs and concluded that increasing the connector cross-sectional area and using a wider shape (ellipse) strongly decreases failure probability [13].

Although a lot of work on FPDs and connector design has been conducted, one might ask if there is a way to check the influence of the connector design for individual geometries with reasonable effort. A possible solution is to automate the design using parametric CAD-modelling, and in a second step FEA, up to a point where one can just import an FPD design and receive a response regarding stress distribution for standard support and standard load cases in automated form. Parametric CAD-modelling has been applied in the field of biomechanics before, for instance, for parametric modelling of a human hand [16], an ear model [17] or the semi-automated generation of bone defects around dental implants [18].

The main goal of this study was to develop an approach for parametric remodelling of the connectors dimension of FPDs. The automated approach was used to generate 3D models, which were used to investigate the influence of connector design on the stress distribution within a 4-unit FPD.

## 2. Materials and Methods

In this study, an algorithm for the automatic adjustment of the connector dimension in n-unit fixed partial dentures (FPD) was developed and implemented in Rhinoceros/Grasshopper (Robert McNeel & Associates, Seattle, WA, USA). The implementation was used to investigate the effect of the connector design on the stress distribution in a 4-unit FPD using finite element analysis (FEA).

The basic geometry of the 4-unit FPD was acquired using an optical scanner (ATOS II SO, GOM GmbH, Braunschweig, Germany) and a master model that was already used in previous studies [19,20,21,22,23,24]. Afterwards, the steps described in Section 2.1 were used to reduce the cross-sectional area from the occlusal, gingival, lingual, buccal or all sides and generate a CAD model of an implant-supported 4-unit FPD that was used for FEA with ANSYS workbench 2022R1 (ANSYS Inc., Canonsburg, PA, USA). The abbreviations used in the next sections denote P: points, L: lines, C: curves, E: planes, M: meshes, B: bounding boxes, R: bounding rectangles, A: areas and d: distances, respectively.

### 2.1. CAD-Modelling

**Figure 1:**
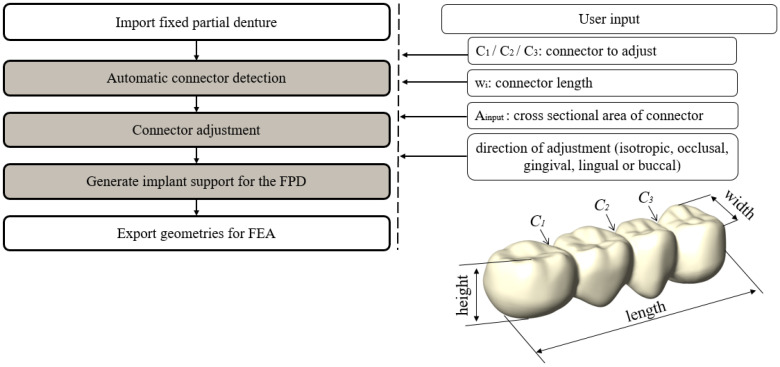
Overview of the proposed algorithm.

An overview of the proposed algorithm can be found in Figure 1. First of all, an STL-File of a 4-unit FPD was imported into Rhino/Grasshopper and a remeshed mesh was computed using Grasshopper’s function Quad-remesh, to allow for a uniform distribution of the mesh vertices *P_i_* along the FPD surface. For this study, the FPD surface was remeshed with 60,000 quads. The remeshed mesh and initial meshes were compared by calculating the Hausdorff distance [25], resulting in a maximum distance deviation of 0.0817 mm and an average deviation of 0.0045 mm from the initial geometry. Afterwards, the positions of the connectors were detected automatically. The mesh vertices *P_i_* were then adjusted according to user-defined parameters. Finally, the mesh was updated using the new vertex positions and supporting implants were placed. Details of these steps are given in the following sections.

#### 2.1.1. Automatic Connector Detection

**Figure 2:**
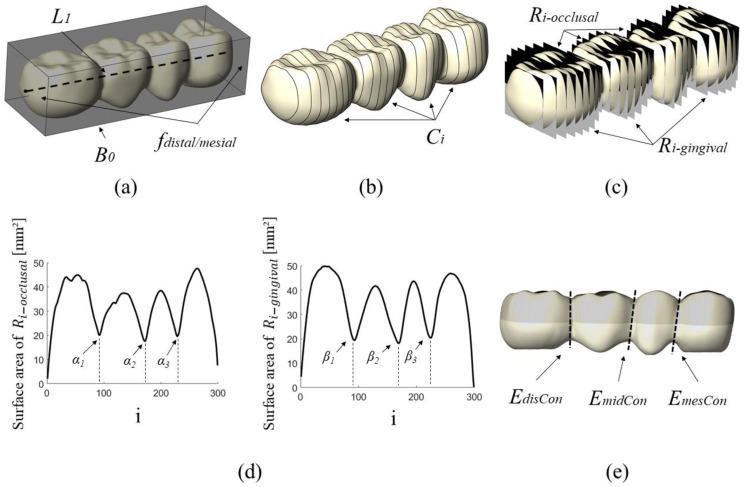
Illustration of substeps for the automatic detection of connectors. Thirty intersecting curves are shown in steps b and c for better visualization, whereas the calculation used 300 intersecting curves. (**a**) Bounding box *B*_0,_ *L_1_* connects the distal and mesial faces *f_distal/mesial_*. (**b**) Intersection curves *C_i_*. (**c**) Bounding rectangles *R_i_*-occlusal and *R_i_*-gingival. (**d**) Surface area plots of bounding rectangles with local minima *α_j_* and *β_j_*. (**e**) Found connector planes *E_disCon_*, *E_midCon_* and *E_mesCon_*.

The connectors of the imported 4-unit FPD were automatically detected using the following steps:

1. Find the smallest bounding box *B*_0_ (with faces *f_occlusal_*, *f_gingival_*, *f_mesial_*, *f_distal_*, *f_lingual_* and *f_buccal_*) for the FPD mesh geometry M_FPD_ using the bounding box function in Grasshopper. Connect the centre of the smallest faces of *B*_0_ (*f_distal_* and *f_mesial_*) with the line *L*_1_, see Figure 2a.
2. Generate 300 equally spaced planes *E_i_* along the line *L*_1_ that are perpendicular to *L*_1_. Find the intersection curves *C_i_* between the planes *E_i_* and the FPD mesh (*M_FPD_*), see Figure 2b.
3. Define the plane *E_mid_* that lies in the middle between the faces *f_occlusal_* and *f_gingival_* of Box *B*_0_. Define the plane *E_mid_*_2_ that lies in the middle between the faces *f_lingual_* and *f_buccal_* of Box *B*_0_.
4. Create bounding rectangles *R_i_* for all intersection curves *C_i_*, with the rectangle’s sides parallel to the faces of the box *B*_0_, see Figure 2c.
5. Divide each bounding rectangle *R_i_* into an occlusal rectangle *R_i_*-occlusal and a gingival rectangle *R_i_*_-gingival_ using *E_mid_*, see Figure 2c.
6. Compute the surface areas (*A_i_*_-occlusal_ and *A_i_*_-gingival_) of each bounding rectangle (*R_i_*_-occlusal_ and *R_i_*_-gingival_). Find the local minima of *A_i_*_-occlusal_ (denoted as *α_j_*) and of *A_i_*_-gingival_ (denoted as *β_j_*), see Figure 2d. The minima are used to define the position of the connectors. As the connectors may be oblique, the minima for the occlusal and gingival sides are calculated separately.

#### 2.1.2. Connector Adjustment

**Figure 3:**
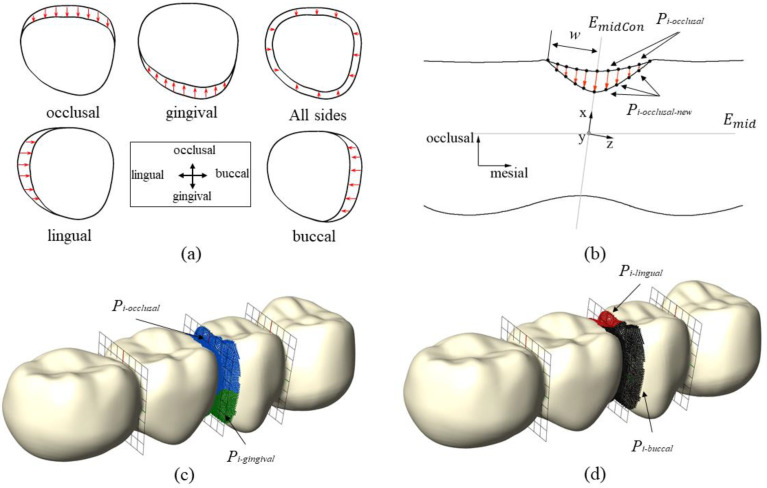
Illustration of substeps for connector adjustment. (**a**) Connectors cross-section for different types of connector shape adjustment. (**b**) Connector adjustment from the occlusal side, shown for a cross-section perpendicular to the lingual-buccal direction. (**c**) Selected points *P_i_*_-occlusal_ (blue) and *P_i_*_-gingival_ (green) for occlusal/ginginval adjustment. (**d**) Selected points *P_i_*_-lingual_ (red) and *P_i_*_-buccal_ (black) for lingual/buccal adjustment.

In this section, the steps for adjusting the connectors are described. The initial cross-sectional areas of the connectors were as follows: the distal connector had an area of 33.1 mm^2^ (with a height of 5.7 mm and length of 6.8 mm), the middle connector had an area of 28.5 mm^2^ (with a height of 5.7 mm and length of 6.0 mm), and the mesial connector had an area of 33.2 mm^2^ (with a height of 5.8 mm and length of 6.8 mm). Due to aesthetic considerations, these connectors were designed in an irregular, oval shape. The connectors were either shrunk evenly in all directions or shrunk from either the occlusal, gingival, lingual or the buccal side. Given that the steps for adjusting the connectors are similar for all directions, only one example is provided in this section to illustrate how the cross-sectional area of the middle connector (*C*_2_) was reduced from the occlusal direction. The different types of adjustment are shown in Figure 3a. To adjust the connector *C*_2_ in the occlusal direction, the following steps were taken:

1. Define *L_o_* as the upper edge of the previously computed bounding rectangle *R_i_*_-occlusal_ at the position of *α_j_* (minimum detected in step 6 in the previous section). Define *L_g_* as the lower edge of *R_i_*_-gingival_ at the position of *β_j_*. Define a plane *E_midCon_* that contains *L_o_* and *L_g_*.
2. Define a Cartesian coordinate system (xyz), whereas the origin is defined by the point, which lies in *E_mid_*, *E_mid_*_2_ (see step 3 in the previous section) and *E_midCon_*. The *x*-axis points from the origin in the direction of the point that is obtained by *E_mid_*_2_ crossing *L_o_*. The *y*-axis lies in *E_midCon_* and points to the buccal side (defined by the previously computed bounding Box *B*_0_). The *z*-axis is chosen perpendicular to the x- and *y*-axis.
3. Compute the distances $d_{\left(i\right)\;}$ of all points of the FPD mesh to the plane *E_midCon_* and select all points that are closer to the plane than w ($d_{\left(i\right)}$ < w), whereas w is a user-defined parameter (w = 1 mm in this study). These selected points are called *P_i-C_*_2_ and their position is altered in the following steps to adjust the connectors.
4. Starting from the point set *P_i-C_*_2_ select all points with x > 0, which are the points close to the occlusal side, see Figure 3c. These selected points are called *P_i-occlusal_*.
5. For each point in *P_i-occlusal_* calculate the distance to the x-y plane (equals *E_midCon_*) and y-z plane. These distances are called $d_{xy\left(i\right)\;}$ and $d_{yz\left(i\right)}.$
6. Compute a new x-coordinate *x_new(i)_* for each point *P_i-occlusal_*, see Figure 3b. *B(t)* is a bézier type function, *x_(i)_* is the old x-coordinate and *k* is a parameter chosen in step 7. The equations were:
  (1) $$x_{new\left(i\right)}=x_{\left(i\right)}-k\;\left(d_{yz\left(i\right)}\;\cdot B\left(d_{xy\left(i\right)}\right)\right)\cdot x_{\left(i\right)}$$
  (2) $$B\left(t\right)=\left({\left(w-t\right)}^{3}\cdot \;w\right)+\left(3t{\left(w-t\right)}^{2}\cdot 0.9w\right)+\left(3t^{2}\left(w-t\right)\cdot 0.1w\right)$$
7. Calculate the cross-sectional area *A*_2_ of connector *C*_2_ and iteratively adjust the value of the parameter *k* in Equation (1) using Galapagos in Grasshopper until *A*_2_ matches the predefined input area value *A_input_*. The optimization was stopped after an accuracy of ± 0.001 mm^2^ was achieved.
8. Reconstruct the FPD mesh using the updated vertices via the Grasshopper function construct mesh.

#### 2.1.3. Implant Support for the FPD

**Figure 4:**
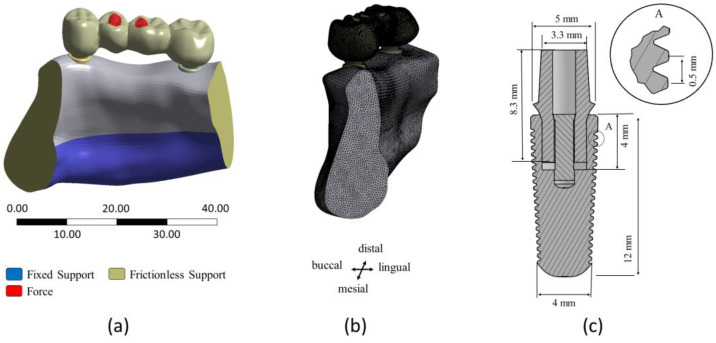
Configuration of the investigated model. (**a**) Boundary conditions. (**b**) Mesh of the FE-model. (**c**) Dimensions of the implant system used in this study.

The 4-unit FPD, measuring 9.0 mm in height, 11.7 mm in width, and 32.5 mm in length, was supported by two conical bone-levelled implants. Each implant measured 12 mm in length, 5 mm in top diameter, 4 mm in bottom diameter, and featured a metric thread design with a thread pitch of 0.5 mm. The abutment measured 8.3 mm in length and had a diameter of 3.3 mm. The abutment screw was modelled without threads to reduce the simulation time. A cement layer with a thickness of 0.1 mm was modelled between the abutment and FPD. The design and dimensions of the implant, abutment and abutment screw are shown in Figure 4c.

### 2.2. Material Properties, Contact Models, Boundary Conditions and Mesh Size

The material properties that were used in the finite element analysis are provided in Table 1. The materials used in this study were modelled with linear elastic and isotropic material models, exempt the transition zone that had a linear graded Young’s modulus. All surfaces between the different components were defined as bonded.

The bone was modelled with a graded transition zone between cortical and cancellous bone. The cortical layer had a thickness of 1.355 mm, and the transition zone had a thickness of 0.29 mm, the Young’s modulus is given in Table 1. Details of the graded bone model and its construction method can be found in Roffmann et al. [26].

**Table 1:** Material properties used in the finite element analysis.

Component	Material	Young’s Modulus [GPa]	Poisson’s Ratio
Implant ^1^	Titanium grade 4	104.5	0.37
Abutment ^1^	Titanium grade 4	104.5	0.37
Implant screw ^1^	Titanium grade 5	114.0	0.33
Cement layer ^2^	Glass ionomer cement	14.3	0.33
FPD ^3^	Zirconium dioxide	210	0.27
Cortical bone ^4^	-	13.7	0.3
Transition zone	-	13.7 to 1.37 (graded)	0.3
Cancellous bone ^4^	-	1.37	0.3

^1^ [27] ^2^ [28] ^3^ [29] ^4^ [30].

The lower surface of the mandibular bone segment was fixed (blue surface in Figure 4a) and both side surfaces were defined as frictionless and supported (yellow surfaces in Figure 4a). In all simulations, a force of 100 N was applied to the occlusal surface of the FPD parallel to the implant axis of both implants (red surfaces in Figure 4a). The force was not applied close to the middle connector as it is often done, because this area changed in our approach and it was the goal to have the same load conditions for all cases. The details of the model can be observed in Figure 4. The FPD was meshed using a mesh size of 0.3 mm. Mesh convergence was checked for different connector geometries. The implant system and bone were meshed with a slightly larger size (0.4 mm implant system, 0.5 mm bone). A total of 1,621,580 tetrahedral elements and 2,374,578 nodes were used in this study. Due to their complex geometries, the FE models were meshed with tetrahedral elements, as shown in Figure 4b.

### 2.3. Evaluation of Results

The developed approach for automatically altering the connector dimensions was applied to a 4-unit FPD. First, the influence of altering the distal connector was analysed, by reducing the connector area by 10% and 20% from all directions, or from either the gingival, occlusal, lingual or buccal direction. The same analysis was repeated for geometries with altered middle connectors and geometries with altered mesial connectors.

The maximum and minimum principal stresses were computed for the 4-unit FPD. For visualization, the FPD was segmented into 200 sections of equal width across its length of 32.5 mm (in the distal–mesial direction), resulting in a section thickness of about 0.16 mm. The highest maximum principal stress and lowest minimum principal stress in each section were chosen for display and plotted along the length coordinate.

**Figure 5:**
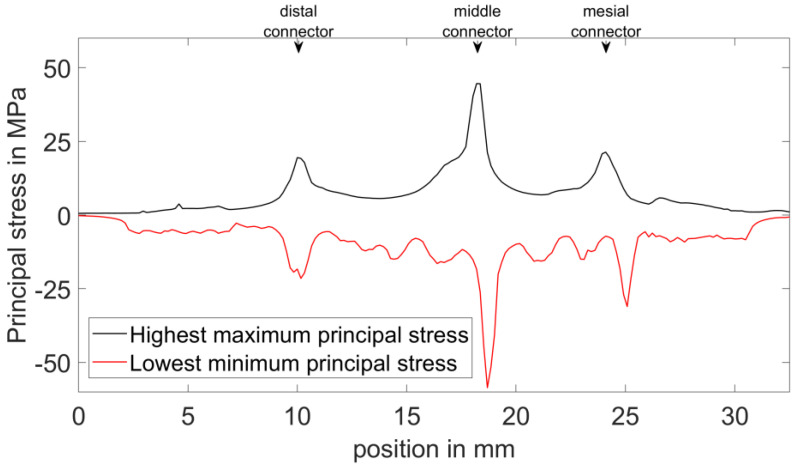
Principal stress behaviour along the length (distal−mesial direction) of the 4−unit FPD without altered connector regions (reference). The main peaks occur at the 3 connector regions.

**Figure 6:**
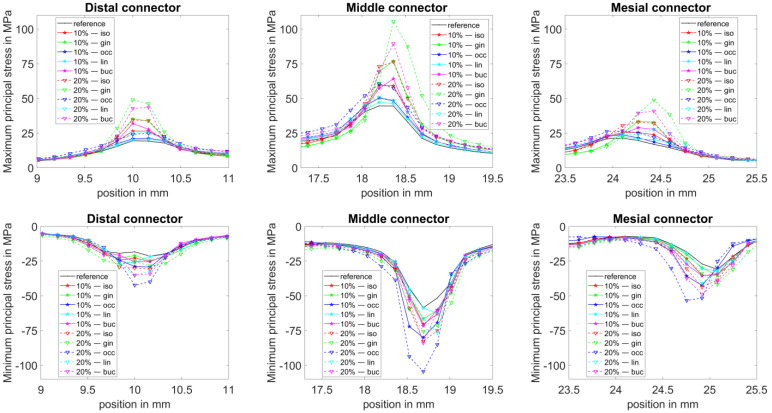
Principal stress behaviour along the length (distal–mesial direction) of the 4-unit FPD for a connector area reduction at either the distal, middle or mesial connector. The connector area was reduced by 10% or 20% from either all (iso), gingival (gin), occlusal (occ), lingual (lin) or the buccal (buc) direction. The stresses are only shown around the connector site where the connector area reduction took place (e.g., the image ‘Distal connector’ shows stresses around the distal connector for geometries with altered distal connectors). The stresses at the non-altered connectors were only mildly affected and are not shown here for clarity.

For clarity, the stresses along the length coordinate are only shown over the full FPD length for the reference design (Figure 5). For the altered geometries (Figure 6), the stresses are only shown around the altered connectors, as the influence on the other connectors was rather low. The low influence can be observed in the data presented in Appendix A.

## 3. Results

In all models, the highest compressive stress (lowest minimum principal stress) was located in the occlusal region of the connectors. The highest tensile stress (highest maximum principal stress) was observed in the gingival region of the connectors. Figure 5 shows the principal stress behaviour along the length of the 4-unit FPD without altered connector regions (method details in Section 2.3).

The stress distribution of the 4-unit FPDs with altered connector areas along the length coordinate is shown in Figure 6. The distal, middle and mesial connectors were altered in separate geometries, with the other connectors remaining unchanged in all cases. A reduction in the cross-sectional area of 10% and 20% was chosen, whereas the reduction was performed either from all directions (iso) or from the gingival (gin), occlusal (occ), lingual (lin) or the buccal (buc) direction. As the main stress changes occur close to the changed connectors, Figure 6 shows only the stresses close to the distal connectors for the geometries with a changed distal connector and vice versa. In Table A1, Table A2 and Table A3, the relative stress change of the maximum principal stress is shown for all connectors, for different reductions of the connector cross-sectional area of the distal (A1), middle (A2) and mesial (A3) connector.

## 4. Discussion

This study aimed to develop an approach for parametric remodelling of the connectors dimension of FPDs and to study the influence of connector design on the stress distribution within a 4-unit FPD.

Overall, the results indicate that the way, how a certain cross-sectional area is modelled, has a large influence on the resulting stresses, depending on the geometric details. A reduction from the occlusal side had the greatest effect on compressive stresses (minimum principal stresses), while a reduction from the gingival side showed the greatest effect on tensile stresses (maximum principal stresses). Given that the tensile stresses become maximal at the gingival side of the middle connector, an area reduction at that side is possibly problematic.

In the literature, in vitro studies also found that the connector design plays an important role in fracture load [6,7,8,9,10,11], which is in line with the general observed stress behaviour in this study. Furthermore, Osman et al. [31] studied the influence of rectangular- and trapezoidal-shaped connectors under different loadings for lithium disilicate and zirconium dioxide three-unit conventional FPD. They found that the base of the connector, regardless of its shape, substantially impacts fracture strength. Arinc [32] performed a finite element analysis for a FPD with three different heights (2 mm, 3 mm and 4 mm). He found that the change in connector height affects the stress distribution in FPDs.

As described in the introduction, Alberto et al. [12] found the radius of the interdental gingival shape to have a significant influence on the stresses in the FPD, whereas the smallest radius led to the highest tensile stresses. The way the connector design is altered in this study is dominated by Equations (1) and (2) presented in Section 2.1.2. These equations were selected to obtain an aesthetically pleasing result and are not optimized for the radius of the interdental shape or minimal stresses. This might be a point for future optimization when the parametric approach is applied in a broader context.

In this study, an axial force of 100 N was applied to the occlusal surface of the FPD. The 100 N are somewhat arbitrary but are often used in dental finite element analysis [33,34,35,36]. The force lies in the range of bite forces for solid food, as reported by Schindler et al. [37] ranging from 20 N to 120 N. Maximum voluntary human bite forces were reported in the review by van der Bilt [38] to be between 306 N ± 42 N and 878 N ± 194 N. Changing the forces in this study to higher values would lead to higher absolute stress values, but the main conclusions of this study are not affected by this point. In a patient-specific analysis, one would instead apply the likely maximum force that can be expected for the patient. Furthermore, the FPD was implant-supported. It is known that the type of support affects the stress in the FPD, whereas more rigid support leads to lower tensile stresses [22].

The current study has certain limitations. The FE models use bonded material interfaces, which is a commonly employed simplification [39,40,41]. However, this simplification may lead to a higher stiffness of the implant and thus a more rigid support. Furthermore, all material properties used in this study were considered as homogeneous, linear and isotropic, apart from the transition zone having a graded Young’s modulus. A further limitation is that the force was directly applied to the occlusal surface, resulting in a simplified contact situation between the FPD and its antagonist compared to previous studies [42]. These simplifications were implemented to reduce the complexity of the FE-model.

The connector adjustment in this study was only performed for one 4-unit FPD. The design parameters, such as wall thickness or crown height, differ between different FPDs. Other designs will have a different stress distribution under load, so the effect of connector adjustment might be slightly different or stress maxima outside of the connectors might arise.

If one for a moment thinks about providing a simulation-based infrastructure to optimize the connector geometries in a dentist’s office, a viable business model is of course mandatory. The computing time needed for the FEA was the biggest time factor and took about 10 min for each simulation on a virtual server using 16 CPU cores (AMD Epyc 7713) and 256 RAM. Licensing an FE software to an individual dentist’s office is probably not realistic, as typically high licensing costs are associated with those licenses. However, this is not a fundamental problem and can be solved in principle. It might be a solution to provide an automated design software as a cloud service, possibly with a specialized licensing model with the FEA software supplier to make it economically viable. Besides the computing itself, a standardization of material constants or even geometry of the supporting bone might be necessary, especially in the first stages, as gathering of individual data can lead to further costs, risks and challenges. However, the current study is still at the basic research level and the focus of this manuscript is on the automation algorithms and not on possible business models.

This study aimed to develop an algorithm to adjust the connector cross-sectional area of a FPD, as a preparation for further automation steps. This goal was successfully achieved, improving the degree of automation in the field of dentistry. Automating the full FDP design including support in a well-controlled way, seems in principle reachable with today’s technology, but still represents a formidable challenge.

## 5. Clinical Relevance

It is generally advisable to avoid low connector area designs, particularly at the gingival side of the middle connector in a 4-unit FPD, due to the susceptibility to high stresses. Besides suitable algorithms a cost-effective method of automation is needed, to be capable to perform a stress analysis of patient-specific FPDs.

## 6. Conclusions

The following conclusions can be drawn from this study:

1. The proposed algorithm enables automatic detection of the connector position and parameterized adjustment of the cross-sectional area of the connectors from various directions.
2. Reducing the connector area from the gingival side has a major influence on the tensile stresses, whereas the middle connector in a 4-unit FPD is most vulnerable regarding a cross-sectional area reduction.

## Data Availability

The data are available on reasonable request.

## Appendix A

**Table A1:** Relative stress change of the highest maximum principal stress at the distal/middle/mesial connector for a changed connector area of the distal connector. iso: reduction from all directions, gin: gingival reduction, occ: occlusal reduction, lin: lingual reduction, buc: buccal reduction.

	Stress Increase in %, When Distal Connector Area 10% Reduced			Stress Increase in %, When Distal Connector Area 20% Reduced		
	Dis. Con	Mid. Con	Mes. Con	Dis. Con	Mid. Con	Mes. Con
Iso	36	0	1	77	0	1
Gin	81	1	−1	152	1	0
Occ	9	0	0	27	−1	−1
Lin	6	1	0	31	1	1
Buc	64	0	1	123	−1	0

**Table A2:** Relative stress change of the highest maximum principal stress at the distal/middle/mesial connector for a changed connector area of the middle connector. iso: reduction from all directions, gin: gingival reduction, occ: occlusal reduction, lin: lingual reduction, buc: buccal reduction.

	Stress Increase in %, When Middle Connector Area 10% Reduced			Stress Increase in %, When Middle Connector Area 20% Reduced		
	Dis. Con	Mid. Con	Mes. Con	Dis. Con	Mid. Con	Mes. Con
Iso	0	33	0	3	71	−1
Gin	0	72	−5	1	137	−6
Occ	1	13	3	4	36	6
Lin	0	6	2	1	14	5
Buc	1	44	0	2	101	−3

**Table A3:** Relative stress change of the highest maximum principal stress at the distal/middle/mesial connector for a changed connector area of the mesial connector. iso: reduction from all directions, gin: gingival reduction, occ: occlusal reduction, lin: lingual reduction, buc: buccal reduction.

	Stress Increase in %, When Mesial Connector Area 10% Reduced			Stress Increase in %, When Mesial Connector Area 20% Reduced		
	Dis. Con	Mid. Con	Mes. Con	Dis. Con	Mid. Con	Mes. Con
Iso	1	−1	19	−1	0	55
Gin	−1	−1	57	0	−1	128
Occ	−1	0	11	0	4	24
Lin	0	4	5	0	3	30
Buc	0	2	36	−1	−1	91
